# Opioid prescribing in general practice: an Australian cross-sectional survey

**DOI:** 10.1186/s12875-022-01783-y

**Published:** 2022-07-08

**Authors:** Sharon Reid, Carolyn Day, Natalie White, Christopher Harrison, Paul Haber, Clare Bayram

**Affiliations:** 1 Speciality of Addiction Medicine, Central Clinical School, Faculty of Medicine and Health, The University of Sydney, NSW 2006 Australia; 2 Drug Health Services, Royal Prince Alfred Hospital, Sydney Local Health District, Camperdown, NSW 2050 Australia; 3 Edith Collins Centre (Translational Research in Alcohol Drugs and Toxicology), Sydney Local Health District, Camperdown, NSW 2050 Australia; 4 Sydney School of Public Health, Faculty of Medicine and Health, The University of Sydney, NSW 2006 Australia; 5 Menzies Centre for Health Policy, Sydney School of Public Health, Faculty of Medicine and Health, The University of Sydney, NSW 2006 Australia

**Keywords:** Opioid prescribing, Prescription opioid, General practice, General practitioner, Benzodiazepine, Chronic non-cancer pain

## Abstract

**Background:**

Prescribed opioid doses > 100 mg oral morphine equivalent (OME) and/or co-prescribing of sedating psychoactive medications increase the risk of unintentional fatal overdose. We describe general practice encounters where opioids are prescribed and examine high-risk opioid prescribing.

**Methods:**

The 2006–2016 BEACH study data, a rolling national cross-sectional survey of randomly selected GPs, was analysed.

**Results:**

Opioid prescribing increased 2006–2007 to 2015–2016, however, this plateaued across the latter half-decade. From 2012–2016 3,897 GPs recorded 389,700 encounters and at least one opioid was prescribed at 5.2%. Opioid encounters more likely involved males, those 45–64 years, concession card holders and the socioeconomically disadvantaged. GPs more likely to prescribe opioids were 55 years or older, male, Australian graduates, and in regional and remote areas. The most common problems managed with opioids involved chronic non-cancer pain. One-in-ten opioid prescribing episodes involved high-risk doses and 11% involved co-prescription of sedating psychoactive medications. Over one-third of GPs provided other (non-pharmacological) interventions at encounters with opioid prescriptions.

**Conclusions:**

Only 5% of GP encounters involved an opioid prescription. Of concern, were: prescribing for chronic non-cancer pain, potentially high-risk opioid encounters where > 100 OME daily dose was prescribed, and/or there was co-prescription of sedating psychoactive medication. However, approximately one-in-three opioid prescribing encounters involved non-pharmacological interventions.

## Introduction

Australia has experienced an increase in pharmaceutical opioid prescribing since 2000 (1–4) linked to increased hospitalisations and accidental death (5). Opioid doses greater than 100 mg oral morphine equivalent (OME)/day significantly increase the risk of unintentional fatal overdose compared with smaller doses, with the threshold for overdose risk starting at greater than 20 mg OME/day (6). Overdose risk is also substantially higher when opioids are used concurrently with benzodiazepines and other sedating psychoactive medications (7). Potential causes of increased opioid prescribing include longer cancer survival periods, an ageing population, and changed patient expectations of living with pain (1, 2, 8).

In 2015, responding to problematic dispensing and prescribing of opioids, the Royal Australian College of General Practitioners (RACGP) produced the ‘Prescribing drugs of dependence in general practice, Part A Clinical Governance framework’ (9) and subsequently released opioid and benzodiazepine prescribing guidelines specifically for general practice settings. Further, in February 2018, the Australian Government Department of Health Therapeutic Goods Administration (TGA) up-scheduled codeine, previously available ‘over-the-counter’ at pharmacies in some formulations (10), to a prescription-only medication. While these recent interventions are likely to have made some impact on prescription-opioid harm, there remains a dearth of knowledge regarding the context in which opioids are prescribed in Australian general practice (GP), especially regarding ‘high-risk’ prescribing, concurrent testing, referrals, and non-pharmacological treatments.

Australian data from 2010–11 found at least one opioid was prescribed/supplied at 4.9% of GP encounters and 5.8% of all GP prescriptions were for opioids, with paracetamol-codeine, oxycodone, and tramadol the most common (11). A large proportion (43.9%) of opioid prescriptions were for chronic non-cancer conditions such as back pain (27.1%), osteoarthritis (9.7%), general multisite pain (6.6%), and migraines (2.0%). Opioids were prescribed most frequently to those aged 55-years and over and in opioid-prescribing consultations, presentations were more likely to be workers’ compensation-related, longer consultations and involved patients with a healthcare card (11). Beyond this, there is little more known about the context of general practice encounters where opioids are prescribed.

In this study, by drawing on historical data from GP encounters where opioids were prescribed, we aimed to further explore the context and patterns of opioid prescribing in Australian general practice with a focus on high-risk opioid prescribing (> 100 OME and/or co-prescribing other sedating psychoactive medications) where the risk of patient harm is greatest. Specifically, we examined: i) rate of encounters in general practice where at least one opioid is prescribed and high-risk opioid prescribing; ii) GP and patient characteristics independently associated with opioid prescriptions; iii) type and rate of opioids prescribed at GP encounters; and iv) problems managed with opioid prescriptions.

## Methods

Data were collected in the Bettering the Evaluation and Care of Health study (BEACH) study, a representative national cross-sectional survey of GP activity in Australia (12, 13). Each year, 1000 randomly sampled GPs recorded the content of 100 consecutive GP-patient encounters on paper recording forms, including details about problems managed, medications, pathology or imaging tests, referrals, and any other clinical or procedural treatments provided. All managements were linked by the GP to the problem being managed. Problems managed were classified according to the International Classification of Primary Care, Version 2 (ICPC-2) (14). All pharmaceuticals were classified using a BEACH classification mapped to the Anatomical Therapeutic Chemical (ATC) Classification (15). Participating GPs provided patients’ date of birth, sex, postcode, concession-card holder, non-English speaking background, Aboriginal and Torres Strait Islander status, and their own age, sex, country of graduation, and postcode of practice. We investigated GPs’ opioid prescribing 2006–16 but focussed on factors associated with prescribing 2012–16 (the last 4 years of the BEACH study).

BEACH was a single-stage cluster-sample study design with the GP as the sampling unit and the GP-patient encounter as the unit of inference. Analyses were undertaken of all encounters where opioids (ATC code N02A) were recorded as prescribed or GP-supplied from April 2012 to March 2016 inclusive. Subgroup analyses were performed on opioid encounters involving other factors that potentially increased the risk of harm.

Data were analysed using univariate methods to describe: i) the encounters involving opioid prescriptions, and ii) rates of high-risk opioid prescribing. Point estimates were calculated using survey procedures in SAS 9.4, which considered the cluster design. Statistically significant difference was determined by non-overlapping 95% confidence intervals (CIs), a conservative estimate of difference which reduces the risk of Type I error while increasing risk of Type II error (16). Multivariate analyses were undertaken to identify independent predictors of opioid prescribing. All GP and patient variables examined at the univariate level were included in a multiple logistic regression, using the survey-logistic procedure in Statistical Analysis Software (SAS) 9.4 accounting for the cluster design.

## Results

**Fig. 1:**
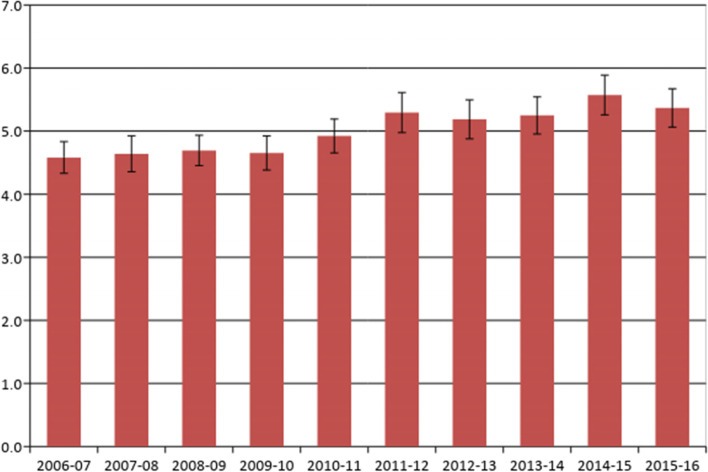
Percent of GP encounter s where at least one opioid prescribed by BEACH year 2006–16 (Error bars = 95% CIs)

There was a significant increase in prescribing of opioids from 2006 to 2016: 4.6% of encounters (95% CI: 4.3–4.8) in 2006–07 and 5.4% (95% CI: 5.1–5.7) in 2015–16, however, this increasing trend plateaued in the latter half-decade (Fig. 1). During 2012–2016, 3,897 GPs recorded details of 389,700 encounters. At least one opioid was prescribed at 5.2% (95% CI: 5.1–5.3) of encounters and most GPs (92.5%) recorded at least one opioid prescription in their encounter activity.

The 24,125 opioids prescribed accounted for 6.0% of all medications recorded, most commonly oxycodone (26.2% of opioids), paracetamol/codeine (24.5%; Table 1). Prescriptions for > 100 mg daily OME made-up 11.1% (Table 1).

**Table 1:** Opioids commonly recorded in Australian general practice, 2012–2016

	**N (% of opioids)**	**Rate per 100 encounters** **(95% CI)**	**Median morphine equivalent dose**^**(a)**^	**Percentage** ** > 100 mg morphine equivalent daily**^**(a)**^
Total opioids	24,125 (100.0)	6.19 (6.02–6.36)	—	11.1% (10.6–11.6)^(b)^
Oxycodone	6,327 (26.2)	1.62 (1.56–1.69)	40 mg (*n* = 4,215)	21.2% (20.0–22.4)
Paracetamol/Codeine	5,919 (24.5)	1.52 (1.46–1.57)	18 mg (*n* = 3,565)	0.0%
Tramadol	3,474 (14.4)	0.89 (0.85–0.93)	30 mg (*n* = 2,479)	0.2% (0.0–0.4)
Buprenorphine	2,405 (10.0)	0.62 (0.58–0.65)	24 mg (*n* = 2,275)	0.0%
Oxycodone/Naloxone	2,075 (8.6)	0.53 (0.50–0.57)	40 mg (*n* = 1,917)	9.5% (8.2–10.8)
Morphine sulphate	1,314 (5.5)	0.34 (0.31–0.37)	60 mg (*n* = 972)	29.3% (26.5–32.2)
Fentanyl	1,161 (4.8)	0.30 (0.27–0.32)	90 mg (*n* = 887)	35.7% (32.6–38.9)
Hydromorphone	340 (1.4)	0.09 (0.07–0.10)	120.8 mg (*n* = 269)	53.1% (47.2–59.1)
Paracetamol/Codeine/ Doxylamine	276 (1.1)	0.07 (0.06–0.08)	18 mg (*n* = 137)	0.0%
Tapentadol	211 (0.9)	0.05 (0.04–0.06)	80 mg (*n* = 194)	30.9% (24.4–37.4)
Codeine phosphate	196 (0.8)	0.05 (0.04–0.06)	9.0 mg (*n* = 122)	0.0%
Morphine hydrochloride	186 (0.8)	0.05 (0.04–0.06)	40 mg (*n* = 180	23.8% (14.4–33.1)

^(a)^ Only reported where complete medication regimen was recorded by the GP

^(b)^ Complete medication regimen was recorded for 17,141 of 24,125 opioid medications. Only opioids recorded at a rate of > 0.5 per 1,000 encounters are included; listed medications account for 99.0% of total opioids

Of the ‘high-potency-opioid’ prescriptions (Fentanyl and Hydromorphone) more than one-third exceeded 100 mg morphine equivalent daily dose. In comparison, of the lower potency opioids prescribed (Paracetamol/Codeine, Paracetamol/Codeine/Doxylamine, and Codeine Phosphate) the median morphine equivalent dose ranged from 9 to 18 mg, and none exceeded 100 mg morphine equivalent dose.

For encounters where an opioid was prescribed, at least one non-pharmacological management (e.g., counselling) was reported at 6,921 encounters (34.1%, 95% CI: 33.0–35.1). Additionally, referrals were provided at 2,905 (14.3%, 95% CI: 13.7–14.9) opioid encounters, imaging-orders at 2,397 (11.8%, 95% CI: 11.3–12.3) and pathology-orders at 2,144 (10.6%, 95% CI: 10.0–11.1). Referral types at all opioid-encounters compared to opioid-problem-linked referrals, were similar (Table 2). The most frequent opioid encounter referrals were to physiotherapists 18.1% (26.8% of opioid-problem referrals) and orthopaedic surgeons 11.4% (15.9%). Opioid-encounter referrals to pain clinic/specialists were 4.2% (6.8% for opioid-problem referrals), to psychologists 3.0% (0.8%), psychiatrists 1.6% (0.4%), oncologists 1.0% (1.1%) and palliative care 0.8% (1.3%). Referrals to drug and alcohol, and mental health nurse/worker/team, were each < 1% referrals (Table 2).

**Table 2:** Referrals at opioid encounters and referrals linked to ‘opioid problem’ 2012–2016

Referral	N (referrals at opioid encounters)	Percentage of ‘opioid encounter’ referrals	N (referrals for ‘opioid problem’)	Percentage of ‘opioid problem’ referrals
**Most frequent referrals (> = 1% of referrals) for opioid problem**				
Physiotherapy	596	18.1	531	26.8
Orthopaedic surgeon	376	11.4	315	15.9
Pain specialist/clinic	137	4.2	134	6.8
Neurosurgeon	122	3.7	112	5.7
Surgeon	136	4.1	72	3.6
Podiatrist/chiropodist	106	3.2	30	1.5
Psychologist	99	3.0	16	0.8
Dentist	98	3.0	80	4.0
Referral (not specified)	85	2.6	51	2.6
Rheumatologist	79	2.4	65	3.3
Clinic/centre	59	1.8	25	1.3
Psychiatrist/Psychiatry clinic	51	1.6	7	0.4
Hospital—referral	49	1.5	30	1.5
Neurologist	48	1.5	27	1.4
Hospital—admission	42	1.3	37	1.9
Emergency Department	45	1.4	29	1.5
Oncologist	34	1.0	22	1.1
Palliative care	27	0.8	25	1.3
Chiropractor	24	0.7	22	1.1
Referral—specialist	22	0.7	19	1.0
**Least frequent referrals (each < 1% of referrals) for opioid problem**				
Referral – Drug & Alcohol	12	0.4	8	0.4
Mental Health—nurse/worker/team	14	0.4	2	0.1
Least frequent referrals—all^a^	354	17.9	693	21.0

^**a**^**Least frequent referrals: 90 other services and specialities, each accounting for < 1% of referrals, including:** Aboriginal health worker, Acupuncture, Aged care assessment, Allergist, Allied health, Antenatal clinic, Audiologist, Audiometry, Breast clinic, Cardiothoracic surgeon,Centrelink,Chemotherapy, Colonoscopy Colorectal surgeon, Community health, Counselling, Diabetes clinic, Diabetes education, Diagnostic – neuro, Diagnostic – Resp, Drug & alcohol, Electrocardiogram, Electroencephalogram, Electromyogram, Endocrinologist, Endoscopy, Exercise physiologist, Family planning, Fertility clinic, Gastroscopy, General practitioner, Geneticist, Geriatrician, Haematologist, Hepatologist, Holter monitor, Home nursing, Home support services, Hospice, Hydrotherapy, Immunologist, Infection specialist, IVF clinic, Masseur, Mental health nurse, Mental health team, Mental health worker, Midwifery services, Monitoring-BP, Mutual support group, Nephrologist, Nerve conduction studies, Nurse, Nursing home, O&G, Occupational therapy, OH&S, OPD, Optometrist, Orthodontist, Orthotist, Osteopath, Paediatrician, Palliative care, Patient support group, Personal trainer, Pharmacist, Physician, Plastic surgeon, Psych clinic, Psychiatrist, Radiation oncologist, Radiologist, Radiotherapy, Rehabilitation, Respiratory physician, Respite care, Sleep clinic, Social worker, Specialist, Speech therapist, Spirometry, Sports medicine practitioner, Stress test, Test-hearing, Vascular surgeon, Vasectomy

Males were more likely to receive an opioid and patients aged 25–44 years and 45–64 were more likely to receive opioids, than were those aged 80 + years (Table 3). Patients more likely to receive an opioid at encounters were those: with a Commonwealth concession card (7.9% of encounters) *c.f*. those without (3.2%); in areas of greatest socioeconomic disadvantage (6.6%) *c.f*. those in areas of highest advantage (4.3%); with an English-speaking background (5.5%) than those of non-English-speaking background (3.5%). Indigenous patients accounted for only 2.9% of opioid encounters but were more likely than non-Indigenous patients to receive opioids (7.8% and 5.3%, respectively). Patients seen previously at the practice comprised 95.7% of opioid encounters, with 4.3% of opioid encounters involving patients new to the practice. These differences persisted after adjustment (Table 3).

**Table 3:** Patient and GP characteristic-specific opioid prescribing rate, 2012–2016

	**Characteristic specific encounters with opioid prescribed** **(n, %) (*n* = 20,307)**	**Encounters in sample (*N* = 389,700)**	**Character-specific proportion of encounters that received an opioid (95% CI)**	**Odds ratio from multivariate logistic regression**^**a**^
**Patient characteristics**				
**Age *(Missing)***	*(152)*	*(3,399)*		*P* < 0.0001
0–14 years	39 (0.2%)	43,752	0.1 (0.1–0.1)	0.02 (0.01–0.03)
15–24 years	587 (2.9%)	30,799	1.9 (1.7–2.1)	0.42 (0.37–0.47)
25–44 years	4,118 (20.4%)	85,293	4.8 (4.6–5.0)	1.16 (1.08–1.25)
45–64 years	7,383 (36.6%)	105,305	7.0 (6.8–7.2)	1.51 (1.41–1.61)
65–79 years	5,074 (25.2%)	79,452	6.4 (6.2–6.6)	0.93 (0.87–0.98)
80 + years	2,954 (14.7%)	41,700	7.1 (6.8–7.4)	Reference group
**Sex *(Missing)***	*(163)*	*(3,418)*		*P* = 0.0059
Male	8,375 (41.6%)	156,312	5.4 (5.2–5.5)	Reference group
Female	11,769 (58.4%)	229,970	5.1 (5.0–5.3)	0.94 (0.91–0.98))
Socio-economic advantage *(Missing)*	*(386)*	*(8,448)*		*P* < 0.0001
High socioeconomic advantage	9,940 (49.9%)	230,055	4.3 (4.2–4.5)	0.82 (0.79–0.86)
Low socioeconomic advantage	9,981 (50.1%)	151,197	6.6 (6.4–6.8)	Reference group
**Patient language background ~ *(Missing)***	*(1,541)*	*(37,716)*		*P* < 0.0001
Non-English speaking	1,098 (5.9%)	31,088	3.5 (3.2–3.8)	0.64 (0.59–0.69)
English speaking	17,668 (94.1%)	320,896	5.5 (5.4–5.7)	Reference group
**Commonwealth concession card holder status** *(Missing)*	*(1,335)*	*(32,201)*		*P* < 0.0001
Card holder	12,743 (67.2%)	161,392	7.9 (7.7–8.1)	2.47 (2.36–2.59)
Non-card holder	6,229 (32.8%)	196,107	3.2 (3.1–3.3)	Reference group
**Indigenous^ Status** *(Missing)*	*(1,545)*	*(37,640)*		*P* = 0.0003
Indigenous	548 (2.9%)	7,017	7.8 (6.8–8.8)	1.30 (1.13–1.50)
Non-Indigenous	18,214 (97.1%)	345,043	5.3 (5.1–5.4)	Reference group
**New to practice status** *(Missing)*	*(284)*	*(5,618)*		*P* < 0.0001
New to practice	851 (4.3%)	28,608	3.0 (2.7–3.3)	0.78 (0.70–0.86)
Seen previously	19,172 (95.7%)	355,474	5.4 (5.3–5.5)	Reference group
**GP characteristics**				
**Age *(Missing)***	*(86)*	*(2,400)*		Not significant (*P* = 0.3329)
< 45 years	4,675 (23.1%)	102,800	4.5 (4.3–4.8)	Reference group
45–54 years	5,703 (28.2%)	110,800	5.1 (4.9–5.4)	1.02 (0.96–1.09)
55 + years	9,843 (48.7%)	173,700	5.7 (5.5–5.9)	1.05 (0.99–1.12)
**Sex *(Missing)***	*(0)*	*(0)*	*(400)*	*P* < 0.0001
Male	12,860 (63.3)	220,500	5.8 (5.6–6.0)	Reference group
Female	7,447 (36.7)	169,200	4.4 (4.2–4.6)	0.82 (0.78–0.87)
**Rurality of GP practice *(Missing)***	*(26)*	*(800)*		*P* < 0.0001
Major city	15,548 (62.1%)	272,700	4.6 (4.5–4.7)	Reference group
Inner regional	5,127 (25.4%)	77,900	6.6 (6.3–6.9)	1.15 (1.09–1.23)
Outer regional/ Remote	2,606 (12.9%)	38,300	6.8 (6.4–7.2)	1.21 (1.11–1.32)
Australian graduate (primary medical degree from Australia) ***(Missing)***	*(101)*	*(1,600)*		P = 0.0060
Yes	13,685 (67.7%)	257,000	5.3 (5.2–5.5)	Reference group
No	6,521 (32.3%)	131,100	5.0 (4.8–5.2)	0.93 (0.88–0.98)

^a^Multivariate Logistic Regression analyses

GPs aged 55 years or older prescribed opioids at a significantly higher proportion of patient encounters (5.7%) and prescribing likelihood was higher among male GPs (5.8% of encounters) than female GPs (4.4%) (Table 3). GPs practising in major cities accounted for the largest volume of opioid encounters (62.1%) but were less likely to prescribe than GPs practising in inner-regional, outer-regional and remote areas. After adjustment, the independent GP-predictors of opioid prescribing were being male, an Australian graduate and practising in inner/outer regional and remote areas (Table 3).

‘Back complaint’ (22.1%), osteoarthritis (10.7%) and generalised pain (7.9%) accounted for more than 40% of the problems managed with an opioid prescription. The management of pain (including ‘chronic pain’) was likely to involve opioids where 70.6% presentations of this ‘specific problem’ was treated with an opioid. Malignant neoplasms accounted for 3.6% of opioids prescribed (Table 4).

**Table 4:** Problems for which opioids were prescribed, 2012–2016

Problem label	Number of problems for which opioid prescribed	Proportion of all opioid problems (95% CI)	Number of times the problem was managed at GP encounters	Proportion of management occasions resulting in opioid script (%) (95% CI)
Back complaint*	4,498	22.1 (21.3–22.8)	11,811	38.1 (36.9–39.3)
Osteoarthritis*	2,192	10.7 (10.2–11.3)	10,819	20.3 (19.3–21.2)
Pain, general/multiple sites (including ‘chronic pain’)	1,613	7.9 (7.4–8.5)	2,286	70.6 (68.5–72.7)
Prescription all*	902	4.4 (4.0–4.9)	11,527	7.8 (7.2–8.4)
Fracture*	776	3.8 (3.5–4.1)	3,723	20.8 (19.4–22.3)
Headache*	591	2.9 (2.6–3.2)	4,303	13.7 (12.5–14.9)
Back syndrome without radiating pain	419	2.1 (1.8–2.3)	1,183	35.4 (32.3–38.6)
Sprain/Strain*	414	2.0 (1.8–2.2)	4,606	9.0 (8.1–9.9)
Injury musculoskeletal NOS	365	1.8 (1.6–2.0)	3,304	11.0 (9.8–12.3)
General symptom/complaint, other	346	1.7 (1.5–1.9)	939	36.8 (33.2–40.5)
Arthritis (excluding OA and rheumatoid)*	340	1.7 (1.5–1.9)	1,999	17.0 (15.2–18.8)
Neurological disease, other	314	1.5 (1.4–1.7)	1,956	16.1 (14.3–17.8)
Rheumatoid arthritis*	269	1.3 (1.2–1.5)	1,902	14.1 (12.5–15.8)
Complication of medical treatment	261	1.3 (1.1–1.4)	1,125	23.2 (20.6–25.8)
Neck syndrome	258	1.3 (1.1–1.4)	1,248	20.7 (18.4–22.9)
Teeth/gum disease	236	1.2 (1.0–1.3)	1,375	17.2 (15.1–19.2)
Other therapeutic procedure/minor surgery NOS	234	1.1 (0.9–1.3)	350	66.9 (61.1–72.6)
Neck symptom/complaint	231	1.1 (1.0–1.3)	1,267	18.2 (15.8–20.7)
Muscle pain	216	1.1 (0.9–1.2)	1,487	14.5 (12.7–16.3)
Shoulder syndrome	209	1.0 (0.9–1.2)	2,393	8.7 (7.6–9.9)
Repair/fixate/suture/cast/ prosthetic device (apply/remove) musculoskeletal	186	0.9 (0.8–1.0)	576	32.3 (28.4–36.1)
Herpes zoster	144	0.7 (0.6–0.8)	926	15.6 (13.2–17.9)
Malignant Neoplasm	729	3.6 (3.3–3.9)	10,641	6.9 (6.3–7.4)
Non-malignant neoplasm chronic problem	9,311	45.7 (44.7–46.6)	207,975	4.5 (4.3–4.6)
Total	20,393	100.0	623,664	3.3 (3.2–3.4)

Co-prescribing of other sedating psychoactive medications occurred at 11.1% (95% CI: 10.6–11.6) of opioid encounters. Benzodiazepine co-prescribing occurred at 7.4% (95% CI: 6.9–7.8) of opioid encounters, Pregabalin 3.3% (95% CI: 3.0–3.6), Quetiapine 0.4% (95% CI: 0.3–0.4), Gabapentin 0.3% (95% CI: 6.9–7.8) and Olanzapine 0.2% (95% CI: 0.1–0.3).

## Discussion

BEACH data across 2006–2016 suggests opioid prescribing increased initially but plateaued in the latter half-decade. Focussing on most recent data from 2012–2016, we found opioids were prescribed in a small proportion of GP encounters (5%), yet most GPs (92%) prescribed opioids at least once in their 100-recorded encounters. One-in-ten were opioids prescribed at high-risk doses (> 100 OME). Additionally, at 11% of opioid encounters there was co-prescription of medications that could increase the risk of harm from opioids.

Comparing this study of BEACH data with other studies concerning prescribed opioids, a recent study of Australian pharmaceutical dispensing data, similarly found that opioid dispensing increased almost four-fold between 1990 to 2014 with the growth in opioid dispensing slowing in the second half of the study period, presumably related to policy and systems level change to reduce prescribed opioid misuse (2). Further, another study (17) estimating global patterns of prescribed opioid consumption found that in 2019 Australia was one of the highest opioid consuming countries (ranked 8th behind the UK, Germany, US, Canada, Spain, Belgium and Denmark) and across 2009 to 2019 there was little change (increase or decrease) in Australia’s prescribed opioid consumption rate in morphine milligram equivalents per 1000 inhabitants per day.

This study, using BEACH data, adds to the literature on opioid prescribing through the provision of detailed contextual information about GP encounters where opioids have been prescribed, including opioid prescribing encounters that can be considered ‘high-risk’ in terms of dose and co-prescribing. This research also highlights potential challenges faced by GPs in their management of patients prescribed opioids. In particular, the low referral rates to clinicians with chronic non-cancer pain (CNCP) expertise, such as Pain and Addiction Medicine specialists and Allied Health professionals, suggest difficulties accessing these clinicians. This inference is supported by the apparent greater risk of opioid prescribing in rural locations where access is likely to be limited but is not conclusive and more work is needed.

This study also calls attention to service accessibility gaps, and potential practice-based and educational interventions around opioid prescribing in general practice. Additionally, the findings indicate that GP opioid encounters are not simple ‘script renewals’ and are more likely very complex and challenging encounters, requiring implementation of non-pharmacological interventions for pain management that are difficult to access through publicly funded clinical services and the prohibitively expensive (for many) private health sector. Previous work suggests about one-third of chronic pain patients are managed with non-pharmacological treatment (18).

During 2012–2016, the prescribing frequency of the different opioid types remained similar to BEACH reporting for 2010–2011 (11), except for oxycodone (alone and in combination with naloxone) becoming the most frequently (35%) prescribed opioid, replacing the paracetamol/codeine combination and potentially attributable to the marketing of oxycodone-naloxone formulations (19). In 2015, the year before BEACH data collection ended, the RACGP guidelines ‘Prescribing drugs of dependence in general practice, Part C1: Opioids’ (20) were released but likely had little time to impact on the GPs participating in the BEACH study. Further, it also probable that the release of guidelines alone, without additional strategies to ensure effective implementation (21), were unlikely to change practice. Also of note, in 2018 after the completion of data collection for this study, low-dose codeine was rescheduled to a prescription only medication and was resisted with concern that codeine users might move to stronger opioids. This, however, has not been apparent in subsequent research (22).

Adjusted analyses indicated those aged 45–64 years were the group most likely to be prescribed an opioid and is consistent with other Australian data ^(4)^. While recent studies report similar rates of opioid prescribing for males and females (23), we found that, after adjustment for other characteristics, male patients were more likely to receive an opioid. This, however, contrasts with other Australian studies where females have been reported to be more likely to be prescription opioid users (23, 24).

GPs in inner-regional, outer-regional and remote areas were more likely to prescribe opioids. Higher prescribing rates for patients in these areas may be influenced by limited access to health services offering non-opioid treatments (25) and fewer opportunities for GP encounters, but warrants further exploration.

Commonwealth concession card holders, and patients with low socioeconomic advantage were more likely to be prescribed opioids. This finding is consistent with other Australian research reporting that socioeconomic disadvantage, complex needs and poor mental and physical health may be linked to longer term opioid use (23, 25), which may exacerbate rather than ameliorate health and wellbeing (26). Indeed, socially disadvantaged populations are overrepresented in cases of unintentional fatal overdose with opioids, typically due to polysubstance use (27).

A significant proportion of people with CNCP are prescribed doses which exceed the overdose risk threshold (6, 23, 28). Prescribers therefore need to be wary of potential comorbidities and contextual factors which may influence the trajectory of a patient’s CNCP, opioid use and overdose risk. Such patients typically require a holistic, multidisciplinary approach to their care, including psychological and physiotherapy services, yet they are likely to experience financial, physical inaccessibility and other barriers to these services (26).

There were no changes in the problems managed with an opioid prescription in 2012–16 to that reported in 2012 (11) and many encounters where an opioid was prescribed were for non-chronic problems. Similar to 2010–2011, 46% of problems managed were CNCP conditions. Likewise specific problems, such as back complaint (22.1%), osteoarthritis (10.7%) and ‘generalised or multisite pain’ (7.9%) remained the most common problems where opioids were prescribed. This consistent trend of prescribing opioids for CNCP is concerning, given the lack of evidence for the efficacy of opioid treatment in CNCP (28).

Our data is limited in that it shows GPs’ intention that the patient takes the medications, but we are unable to verify if this occurred. Additionally, the cross-sectional nature of the study means that information about other relevant health conditions, not managed at the encounter, were not recorded. Further, some GP data forms had missing data but was generally less 1% (documented in data tables). However, social desirability bias was unlikely given the de-identified nature of BEACH data collection, and the impact of recording errors were minimized given the structured BEACH data forms and data checking processes. As BEACH data collection ended in 2016, it is possible that trends in opioid prescribing may have changed somewhat in recent years, however, this study examining a representative sample of Australian GPs prescribing opioids, provides insights into potentially ‘high-risk’ opioid prescribing by dose and the co-prescription of other high-risk medications.

## Conclusions

During the study period (2006–2016) opioid prescribing increased initially but later plateaued with no significant change across 2012–2016 which may reflect the effectiveness of educational interventions and the introduction opioid prescribing guidelines specifically for GPs. Additionally, at over one-third of opioid encounters, GPs provided other (non-pharmacological) interventions such as counselling, referrals, and investigations. However, high-risk opioid encounters where large OME doses were prescribed and/or the co-prescribing of sedating psychoactive medications, along with opioid prescribing for CNCP, remain a concern. This study importantly highlights the potential challenges faced by GPs managing patients prescribed opioids. Difficulties accessing CNCP expertise, and the need for interventions specifically focussing on the indications for opioid prescribing in CNCP and high-risk opioid-prescribing encounters warrant further investigation.

## Data Availability

Information about access to the BEACH dataset supporting the conclusions of this article can be obtained from the data custodian Dr Chris Harrison (christopher.harrison@sydney.edu.au).

## Abbreviations

AIHW: Australian Institute of Health and Welfare
ATC: Anatomical Therapeutic Chemical
BEACH: Bettering the Evaluation and Care of Health
CNCP: Chronic Non-Cancer Pain
CIs: Confidence Intervals
GP: General Practitioner
GPs: General Practitioners
OME: Oral Morphine Equivalent
RACGP: Royal Australian College of General Practitioners
SAS: Statistical Analysis Software
TGA: Therapeutic Goods Administration
